# Proximal tibial anatomical axis and anterior tibial cortex‐based measurements of posterior tibial slope on lateral radiographs differ least from actual posterior tibial slope—A biomechanical study

**DOI:** 10.1002/jeo2.70108

**Published:** 2024-12-11

**Authors:** Christian Peez, Caroline Waider, Adrian Deichsel, Thorben Briese, Lucas K. Palma Kries, Elmar Herbst, Michael J. Raschke, Christoph Kittl

**Affiliations:** ^1^ Department of Trauma, Hand and Reconstructive Surgery University Hospital Münster Münster Germany

**Keywords:** biomechanics, measuring techniques, posterior tibial slope, radiographs

## Abstract

**Purpose:**

To compare different measurement techniques of the posterior tibial slope (PTS) on lateral radiographs with the actual in situ PTS and evaluate the effect of tibial malrotation and image section length.

**Methods:**

Actual PTS was measured on eight fresh‐frozen tibiae using a portable 6‐axis measuring arm with an accuracy of ±0.01°. True lateral radiographs were taken in the neutral position and after applying 10/20/30° internal/external rotation (IR/ER) and 5/10/15° varus/valgus rotation. The PTS was measured radiographically using five different reference axes: anterior tibial cortex (ATC), anatomical tibial axis, proximal tibial anatomical axis (PTAA), posterior tibial cortex (PTC) and fibular shaft axis (FSA).

**Results:**

The ATC and PTAA methods showed the lowest deviation from the actual PTS, while the PTC method showed the highest difference of 5.5 ± 1.5° (medial) and 7.1 ± 1.8° (lateral) among all tested methods (*p* < .001). The PTAA technique showed a 1.9 ± 1.4° (medial) and 2.9 ± 1.8° (lateral) difference from the actual slope (n.s.). ER caused the PTS to increase 0.7 ± 2.0° (10° ER, n.s.) to 3.4 ± 2.1° (30° ER, *p* < .05), whereas IR caused the PTS to decrease 1.6 ± 1.3° (n.s) to 4.1 ± 1.7° (*p* < .05) when comparing to the PTAA method for the neutral position. Varus and valgus rotation showed the highest deviation from the neutral rotation at 15° valgus (3.1 ± 2.1°, n.s.).

**Conclusion:**

Tibial slope measurements have a high degree of variability between different measurement methods, while the ATC and PTAA methods showed the least deviation from the actual PTS measured in this in vitro model. Malrotation resulted in a severe distortion of the PTS values, which may alter preoperative planning and intraoperative results. Therefore, radiographic PTS measurements may be contrasted with more objective, reproducible and reliable measuring methods.

**Level of Evidence:**

There is no level of evidence as this study was an experimental laboratory study.

**Abbreviations**
ACL: anterior cruciate ligament
ACLR: anterior cruciate ligament reconstruction
ATA: anatomical tibial axis
ATC: anterior tibial cortex
ER: external rotation
FSA: fibular shaft axis
IR: internal rotation
PTAA: proximal tibial anatomical axis
PTC: posterior tibial cortex
PTS: posterior tibial slope
SD: standard deviation

## INTRODUCTION

An increased posterior tibial slope (PTS) has recently gained interest as a cause of recurrent instability following anterior cruciate ligament reconstruction (ACLR) [3, 10, 37, 44]. Recent biomechanical studies have shown that a steep PTS causes increased anterior tibial translation [1, 15] and thus loading on the ACL graft [5], so an increased PTS is considered an important predictor of recurrent ACL instability [17, 37, 44]. Accordingly, a clinical study evaluating adolescent and adult patients undergoing hamstring tendon autograft ACLR has identified a PTS > 12° as a critical threshold value for an increased risk of graft failure [44], which was maintained at 20‐year follow‐up and even more pronounced for adolescents [37]. Consistent with these clinical findings, recent biomechanical studies have shown that slope‐reducing osteotomies decrease anterior tibial translation and the load on the ACL graft [5, 24]. Therefore, in patients with a high PTS, combined ACLR, and slope‐reducing osteotomy have become a treatment option with promising clinical short‐term results, especially in revision surgery [3, 10, 42, 45].

Similar to coronal plane osteotomies in which patient outcome is dependent on preoperative planning and correct indications [21, 23, 39], an accurate and reproducible measurement of PTS becomes crucial for clinical decision‐making in ACL surgery [7, 32]. However, several different techniques for assessing the PTS have been reported [13, 25, 34, 43], but no standard imaging technique [9, 13, 22, 26, 43] or reference axis [4, 8, 30, 35] has been established. For example, the use of the anterior tibial cortex (ATC) or the posterior tibial cortex (PTC) leads to a ± 5° difference in the measured PTS values [8, 30], which may alter patient selection and preoperative planning. In addition, malrotation of the tibia and the length of the radiograph could lead to a PTS measurement error of up to 14° [26], so the interpretation and comparison of reported PTS values and thresholds in the current literature is limited.

Therefore, the aim of the present study was to compare different measurement techniques of the PTS on lateral radiographs with the actual in situ PTS and evaluate the effect of tibial malrotation and image section length. It was hypothesized that (1) the lateral and medial PTS measured in a lateral radiograph using the anatomical axis will correlate best with the actual PTS and that (2) internal/external rotation (IR/ER) and varus/valgus rotation as well as (3) the length of the radiograph will alter the results.

## MATERIALS AND METHODS

Eight fresh‐frozen human cadaveric tibiae with a mean age of 80.9 ± 7.2 years (three female, five male) from a local tissue bank were used for this study. The knee specimens were dissected and biomechanically tested with the permission of the Institutional Ethics Committee (File number 2022‐198‐f‐S).

### Specimen preparation

**Figure 1:**
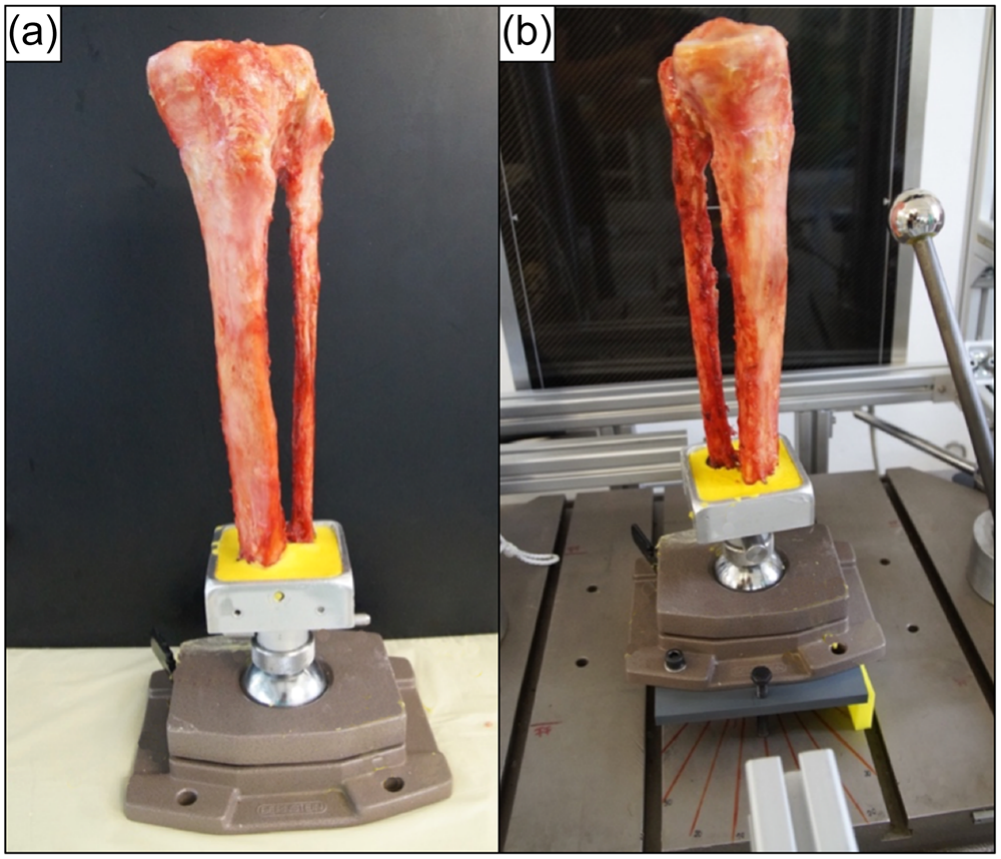
Photograph of the test setup for a left tibia. (a) The specimen was fixed in a custom‐designed vise fixture using polymethylmethacrylate, allowing unconstrained positioning. (b) Using a rotary/tilting plate, each specimen could be rotated internally/externally and varus/valgus tilted.

The cadaveric tibiae were stored at −20 C° and thawed for 24 h at room temperature prior to dissection. After the removal of all soft tissue, the tibiae were cut at the transition from the distal metaphysis to the diaphysis. Then, a stainless steel rod with a diameter of 10.0 mm was inserted toward the proximal tibia representing its anatomical axis. The distal end of the tibia was cemented in a custom‐designed vise fixture using polymethylmethacrylate (Figure 1a), which was fixed on a rotary/tilting plate, allowing unconstrained positioning of the specimens prior to testing (Figure 1b).

### In situ measurements of PTS

**Figure 2:**
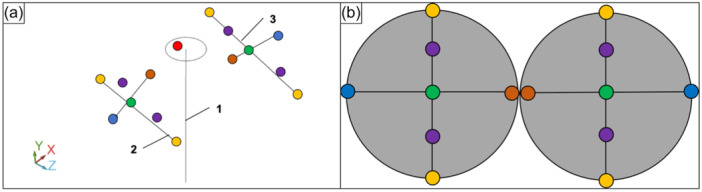
Illustration of the measurement of the actual posterior tibial slope. (a) Screenshot of the metrology software (PC‐DMIS 2019 R1, Hexagon Metrology GmbH) from a single specimen while data processing of a left tibia. The circumference of the stainless‐steel rod was digitized and approximated as the anatomical tibial shaft axis (1). Each dot represented the border of the medial and lateral tibial plateau, which were approximated to lines in order to represent the sagittal inclination of the lateral (2) and medial tibial plateau (3). (b) Schematic illustration of the measurement points for the digitization of the medial and lateral tibial plateau. Each dot represented a specific anatomic landmark: the centre of the tibial plateau (green), central border of the tibial plateau (orange), peripheral border of the tibial plateau (blue), anterior and posterior border of the tibial plateau (yellow) and anterior and posterior quarter of the tibial plateau (violet).

A portable 6‐axis measuring arm (Absolute Arm 8320‐7, Hexagon Metrology GmbH) with an accuracy of ±0.019 mm was used for assessing the actual PTS of the medial and the lateral tibial plateau. By using a touch trigger probe, the anatomical axis of the tibia as well as the medial and lateral tibial plateau, were digitalized using metrology software (PC‐DMIS 2019 R1, Hexagon Metrology GmbH), which can create an x‐y‐z coordinate system of the tibia [18] and precisely measure objects in a 3D space (Figure 2). Following the fixation of the tibia in the custom‐designed vise fixture, six points were digitized circumferentially at the proximal and distal end of the inserted steel rod in order to reference the centre of the rod as the anatomical tibial shaft axis. Then, the medial and lateral tibial plateau were digitized using seven points each, in order to determine the inclination of the respective tibial plateau in the sagittal plane. Finally, the anatomical axis of the tibia and the inclination of the tibial plateau in anteroposterior orientation were approximated by the metrology software as lines, and the actual PTS of the medial and lateral tibial plateau was calculated as the angles between each respective axis.

### Radiographic measurement of PTS

**Figure 3:**
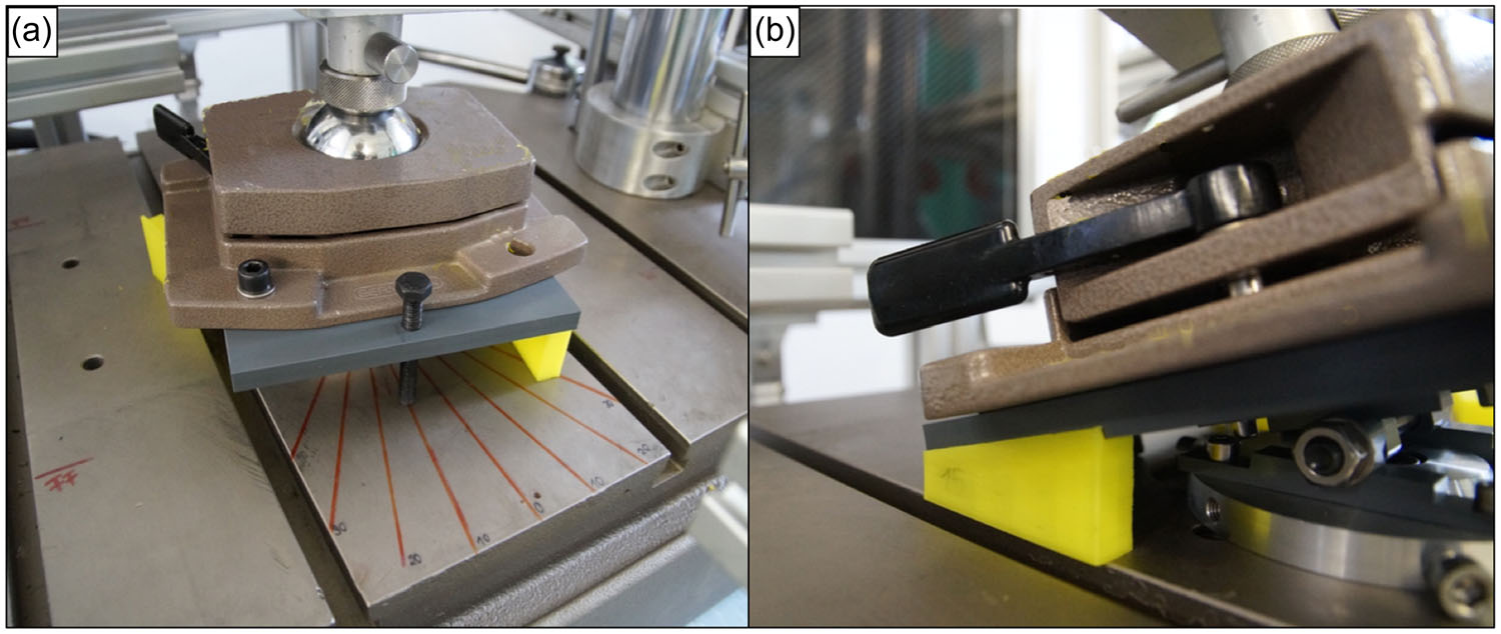
Photograph of the rotary/tilting plate. Each specimen was fixed in 0° external/internal rotation and 0° varus/valgus rotation based on true lateral radiographs. (a) The rotatory component allowed the tibia to be positioned in 10°, 20° and 30° internal and external rotation. The locking screw prevented the plate from tilting and served as a pointer for correct positioning. The figure shows the plate in 10° internal rotation. An internal rotation of 10° is shown as an example. (b) A 3‐dimensional printed polyethylene wedge was placed under the rotary/tilting plate allowing the tibia to be positioned in 5°, 10° and 15° varus and valgus rotation. A valgus rotation of 15° is shown as an example.

An x‐ray source was placed 1 m from the tibia. Based on true lateral radiographs, each specimen was fixed in a neutral position (0° IR/ER and 0° varus/valgus rotation), which was defined (1) by a perpendicular orientation of the x‐ray beam to the tibial shaft axis and (2) by a perfect superimposition of the medial and lateral posterior tibial condyles. From this neutral position, each specimen could then be rotated internally/externally by 10°, 20° and 30° (Figure 3a) or tilted by 5°, 10° and 15° varus/valgus rotation (Figure 3b) using the rotatory/tilting plate to produce malrotated radiographs of the tibia. A lateral radiograph was taken in each position.

**Figure 4:**
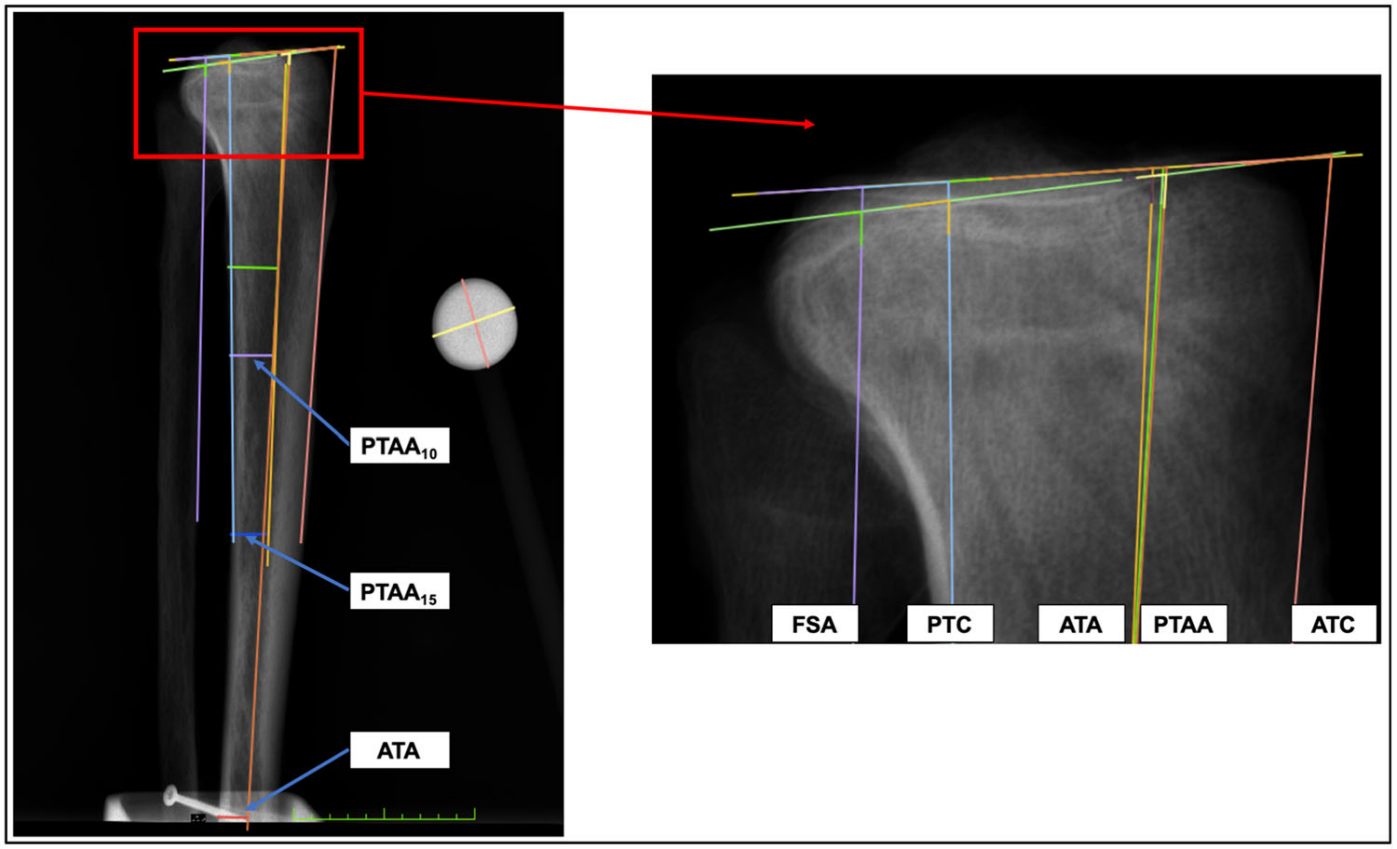
True lateral radiograph of a left tibia. The posterior tibial slope was defined as the angle between the line perpendicular to six different anatomical references and the line tangent to the medial and lateral tibial plateau. ATA, anatomical tibial axis (orange), line connecting the midpoints of the outer cortical diameter at 5 cm distal and maximum length of the tibia; ATC, anterior tibial cortex (red); PTAA_10_, proximal tibial anatomical axis (yellow), line connecting the midpoints of the outer cortical diameter at 5 and 10 cm distal to the tibial tuberosity; PTAA_15_, proximal tibial anatomical axis (green), line connecting the midpoints of the outer cortical diameter at 5 and 15 cm distal to the tibial tuberosity; PTC, posterior tibial cortex (blue); FSA, fibular shaft axis (violet).

The PTS was defined by the angle between a line perpendicular to different anatomical reference axes and the line tangent to the medial and lateral tibial plateau. The following anatomical reference axes were used: ATC, proximal tibial anatomical axis (PTAA), anatomical tibial axis (ATA), PTC and fibular shaft axis (FSA). The ATC and PTC were defined as the connecting line of two points on the anterior/posterior cortex of the proximal tibia at 5 and 15 cm distal to the tibial plateau [8, 30]. The PTAA was defined as a line connecting the midpoints of the outer cortical diameter at 5 and 10 cm (PTAA_10_) [13], such as 5 and 15 cm (PTAA_15_) [4, 11] distal to the tibial tuberosity. A mid‐diaphyseal line connecting the midpoints of the outer cortical diameter at 5 cm distal to the tibial tuberosity and the maximum length of the tibia formed the ATA [35], which represented full‐length PTS measurements. Finally, tangent to the anterior cortex of the fibular shaft segment formed the FSA [8] (Figure 4).

The radiographic and in situ slope measurements were performed by a senior orthopaedic surgeon, who specialized in knee surgery (C.K.).

### Data processing

After assessing the PTS in situ and radiographically, the values of the actual PTS were subtracted from the values of PTS after radiographic measurement. Therefore, the deviation of the radiographically measured PTS values from the actual PTS could be determined for the respective tibial plateau depending on the used anatomical references, the degree of IR/ER and valgus/varus rotation.

### Statistical analysis

Statistical analysis was performed using PRISM (Version 9, GraphPad Software). Descriptive data is presented in terms of mean value ± standard deviation (SD). The normality of data distribution was approved by the Shapiro–Wilk Test. A two‐way repeated‐measures analysis of variance with Geisser‐Greenhouse correction was used to determine any statistically significant differences between the actual PTS and the different measuring techniques on lateral radiographs in the neutral and malrotated tibiae. Post hoc Tukey correction was performed to account for multiple tests. Pearson correlation and linear regression were obtained to calculate the interrelationship between the actual PTS and the different measurement methods. Correlation coefficients between 0.40 and 0.69 were defined as ‘moderate positive correlation’, between 0.70 and 0.89 were defined as ‘strong positive correlation’, and between 0.90 and 1.00 were defined as ‘very strong positive correlation’ [38]. *p* < .05 were considered statistically significant.

An a‐priori power analysis was performed using G*Power‐2 software (University Düsseldorf, Düsseldorf, Germany) [14]. Based on means and SDs from previous studies [13, 43], it was assumed that a sample size of 8 would allow the identification of changes in PTS of 3° with an SD of 2° (effect size/Cohens *d* = 1.5), with 95% power, at the significance level of *p *= .05.

## RESULTS

The mean PTS values of the different measurement techniques are summarized in Table 1.

**Table 1:** Mean posterior tibial slope values of the different measurement techniques ± standard deviation in neutral rotation.

Measurement technique	Medial tibial plateau	Lateral tibial plateau
In‐situ measurement	9.7 ± 3.4	11.5 ± 2.1
ATC	8.9 ± 2.9	9.6 ± 2.2
ATA	7.6 ± 2.4	8.3 ± 1.9
PTAA_10_	7.8 ± 2.6	8.5 ± 1.5
PTAA_15_	6.8 ± 2.5	7.4 ± 2.2
PTC	5.9 ± 2.6	7.1 ± 3.0
FSA	5.8 ± 2.4	6.3 ± 2.9

Abbreviations: ATA, anatomical tibial axis; ATC, anterior tibial cortex; FSA, fibula shaft axis; PTAA_10_, proximal tibial anatomical axis_10_; PTAA_15_, proximal tibial anatomical axis_15_; PTC, posterior tibial cortex.

### Effect of anatomical reference selection

**Figure 5:**
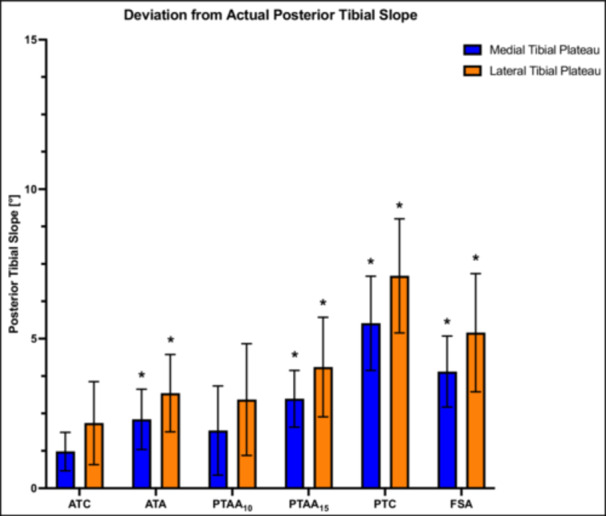
Deviation (in°) of the radiographically assessed posterior tibial slope from the actual values in the neutral position of the tibia (0° external and internal rotation, 0° varus and valgus rotation) for the medial and lateral tibial plateau. ATA, anatomical tibial axis; ATC, anterior tibial cortex; FSA, fibula shaft axis; ns, not significant; PTAA, proximal tibial anatomical axis; PTC, posterior tibial cortex. *p < .05.

Regardless of the measuring technique, evaluation of the PTS on the medial plateau showed less deviation from the actual PTS compared to the lateral PTS (*p* < .05). Accordingly, the values of lateral PTS were at least 1.3 ± 0.3° higher than the values for the medial tibial plateau. The ATC method showed the lowest deviation (n.s.) from the actual PTS of 1.2 ± 0.6° for the medial and 2.2 ± 1.3° for the lateral tibial plateau. Conversely, the PTC had a difference of 5.5 ± 1.5° (medial) and 7.1 ± 1.8° (lateral), which was the highest (*p* < .05) among all tested methods. The PTAA or ATA method, which refers to the midportion of the tibia, showed values in the range of the ATC and PTC methods. Using the PTAA_10_ technique, there was low deviation (n.s.) from the actual PTS of 1.9 ± 1.4° for the medial and 2.9 ± 1.8° for the lateral tibial plateau, while the PTAA_15_ had a difference of 2.9 ± 0.9° (medial) and 4.1 ± 1.6° (lateral) from the actual slope (*p* < .05). Additionally, the ATA method showed significant deviation from the actual PTS of 2.3 ± 1.0° for the medial and 3.1 ± 1.3° for the lateral tibial plateau (Figure 5).

### Effect of internal/external and varus/valgus rotation

**Figure 6:**
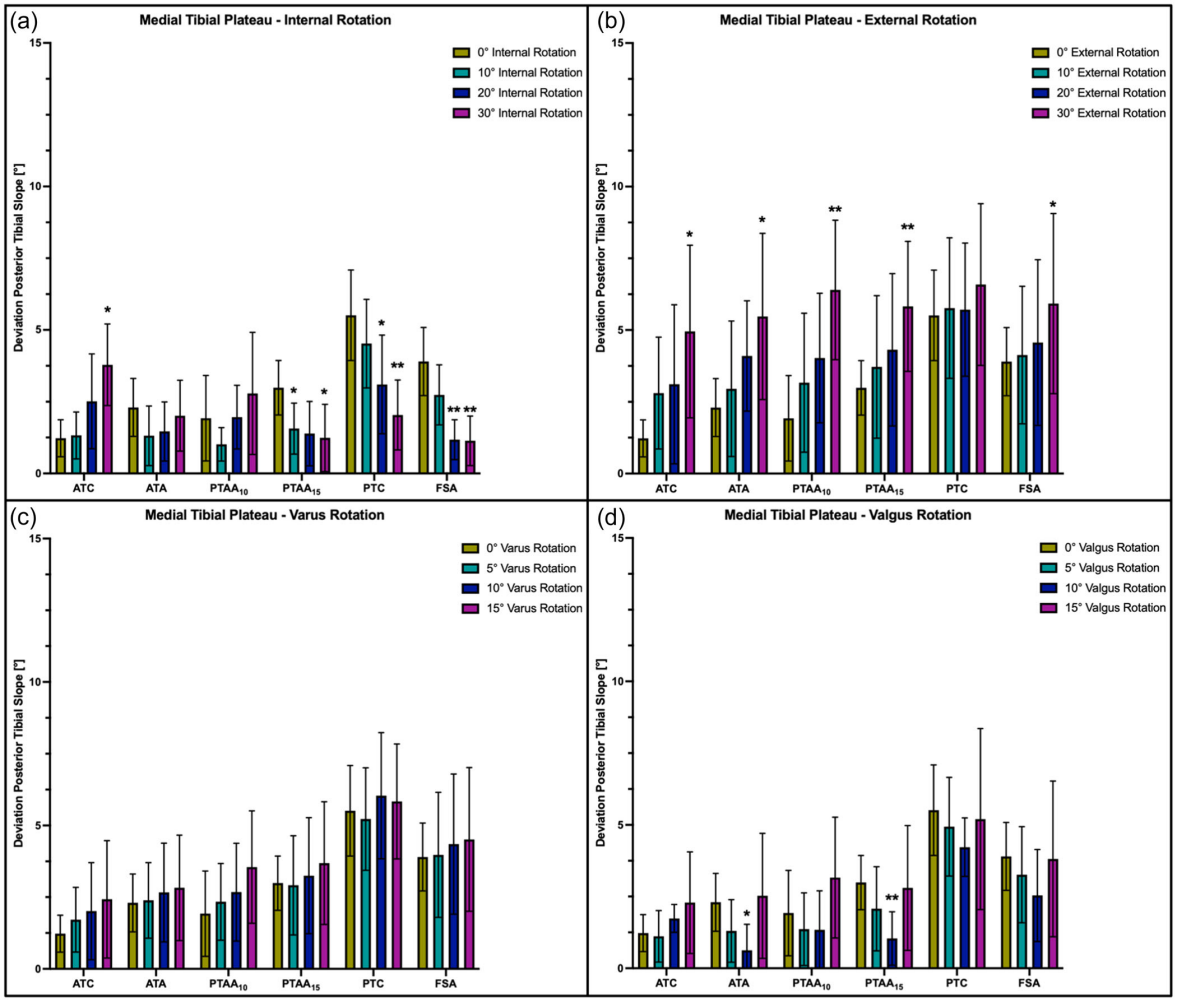
Deviation (in°) of the radiographically assessed posterior tibial slope from the actual values of the medial tibial plateau depending on the degree of tibial malrotation. (a) Internal rotation. (b) External rotation. (c) Varus rotation. (d) Valgus rotation. ATA, anatomical tibial axis; ATC, anterior tibial cortex; FSA, fibula shaft axis; ns, not significant; PTAA, proximal tibial anatomical axis; PTC, posterior tibial cortex. *p < .05, **p < .01.

**Figure 7:**
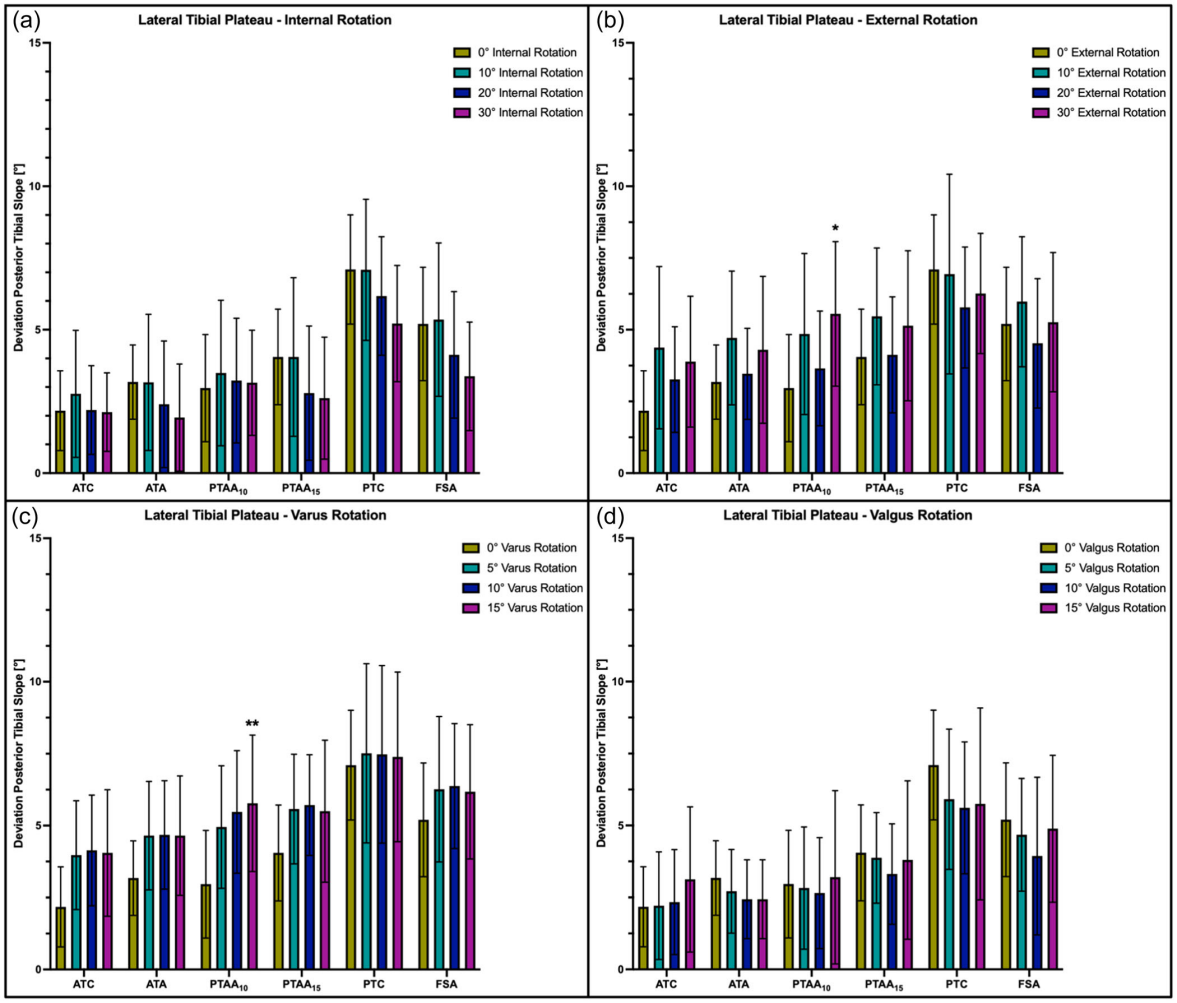
Deviation (in°) of the radiographically assessed posterior tibial slope from the actual values of the lateral tibial plateau depending on the degree of tibial malrotation. (a) Internal rotation. (b) External rotation. (c) Varus rotation. (d) Valgus rotation. ATA, anatomical tibial axis; ATC, anterior tibial cortex; FSA, fibula shaft axis; ns, not significant; PTAA, proximal tibial anatomical axis; PTC, posterior tibial cortex. *p < .05, **p < .01.

Regardless of the side of the tibial plateau, the measured slope values reacted with a reciprocal behaviour to tibial malrotation. External and varus rotation caused the PTS to increase, whereas internal and valgus rotation resulted in a decrease in the slope values. For example, ER resulted in an increased PTS of 0.7 ± 2.0° (10° ER, n.s.) to 3.4 ± 2.1° (30° ER, *p* < .01)), whereas IR led to a decreased PTS of 1.6 ± 1.3° (10° IR, *p* < .05) to 4.1 ± 1.7° (30° IR, *p* < .05) when comparing the PTAA_10_ method to the neutral position (Figure 6). Varus rotation led to the highest deviation from the neutral position for the PTAA_10_ method, which resulted in an increase of 1.6 ± 1.7° (15° varus rotation, n.s.) and 2.8 ± 1.8° (15° varus rotation, *p* < .01) for the medial and lateral PTS, respectively. When comparing to neutral position, valgus rotation caused the highest decrease of the medial PTS for the PTAA_15_ (1.9 ± 0.8° in 10° valgus rotation, *p* < .01) and ATA method (1.7 ± 0.8° in 10° valgus rotation, *p* < .01), whereas the highest decrease in lateral PTS was shown for the PTC technique at 15° valgus rotation (1.4 ± 1.2°, n.s.). When comparing the PTAA_10_ and ATC, varus and valgus rotation showed the highest deviation from the neutral rotation at 15° valgus (3.1 ± 2.1° and 2.3 ± 1.8°) and 15° varus (3.5 ± 1.9° and 2.4 ± 2.1°), which was not significant (Figure 7).

### Effect of radiograph length

**Figure 8:**
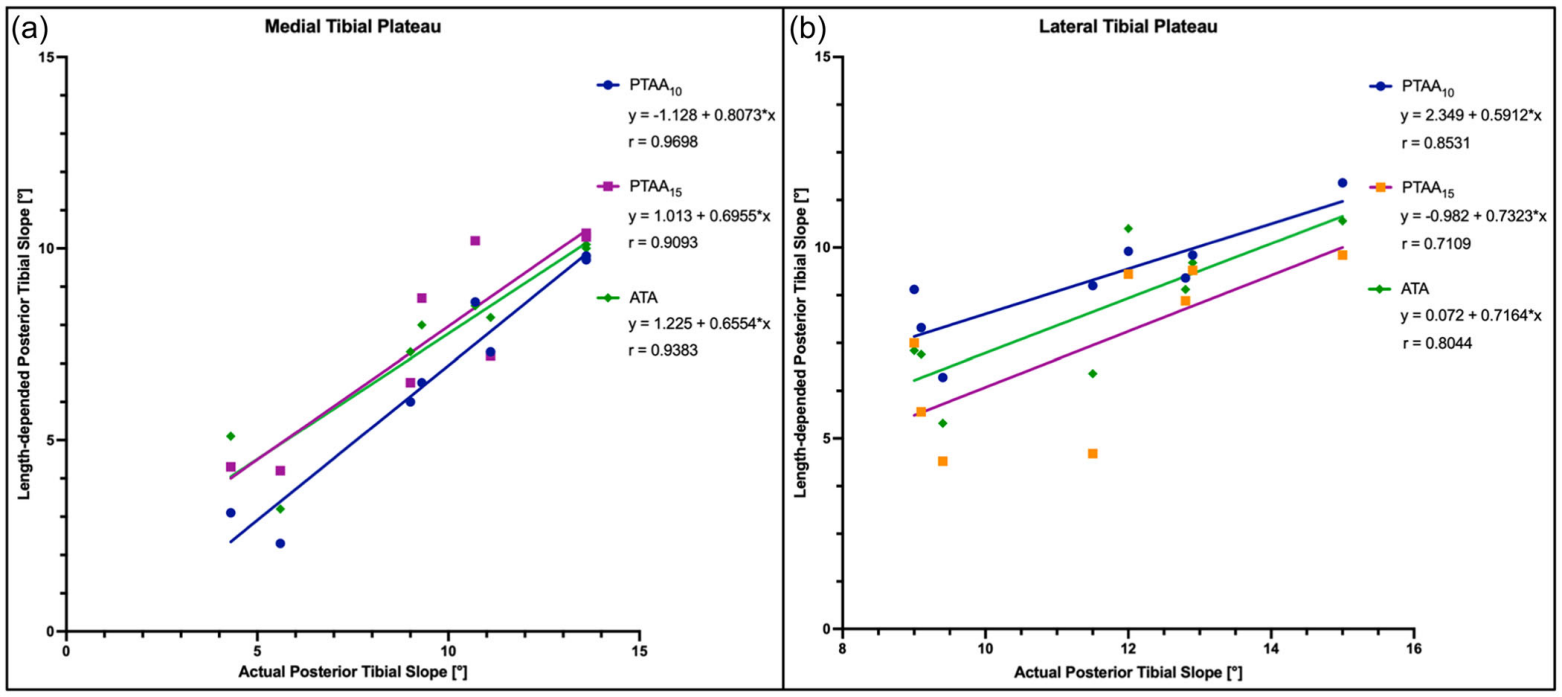
Pearson correlation analysis and linear regression showed a strong and significant linear relationship between the actual posterior tibial slope and measurements based on a mid‐diaphyseal reference axis of different lengths. (a) Medial tibial plateau. (b) Lateral tibial plateau. ATA, anatomical tibial axis (green); PTAA_10_, proximal tibial anatomical axis (blue); PTAA_15_, proximal tibial anatomical axis (orange).

For the medial tibial plateau, there was no significant difference between the PTS values measured with full‐length (ATA: 7.5 ± 2.3°) and a shorter tibial axis (PTAA_10_ 6.6 ± 2.8° and PTAA_15_: 7.7 ± 2.5°, n.s.). Accordingly, the linear correlation showed a very strong positive relationship between the actual PTS and ATA and the PTAA technique (*r* at least 0.90; *p* < .001). When measuring the lateral PTS, the ATT method (8.2 ± 1.9°) also showed no significant difference to the PTS values based on short tibial axis measurements (PTAA_10_ 9.1 ± 1.4° and PTAA_15_ 7.4 ± 2.2°, n.s.). For this, a lower but still strong positive and significant correlation was found between the actual PTS and the ATA and PTAA method (*r* at least 0.71, *p *< .01) (Figure 8).

## DISCUSSION

The most important finding of the present study was that tibial slope measurements using lateral knee radiographs showed high variability and deviation compared to the actual PTS, with the ATC and PTTA_10_ methods differing least from the in situ measured slope values. Regardless of its length, measurements with a mid‐diaphyseal reference axis showed a high accuracy compared to the actual PTS. However, tibial malrotation altered the slope measurements, with external and varus rotation overestimating and internal and valgus rotation underestimating the slope values, so preoperative planning should not be performed on highly malrotated radiographs.

The high degree of variability in slope measurements has been mainly attributed to the measurement method, acquisition technique and imaging modality [2, 31], although further confounders, such as the length of the reference axis [13, 33] and tibial malpositioning [6], have been described. In 2014, Faschingbauer et al. [13] have shown, that the slope values are increasingly overestimated with decreasing PTAA length on plain radiographs. A short PTAA of 5 cm resulted in a 2.9° increase of the PTS compared to the mechanical tibial axis, whereas in the present study, the length of mid‐diaphyseal reference axes (PTAA_10_ = 5 cm, PTAA_15_ = 10 cm, ATA = maximum tibial length) did not a significant deviation from the actual PTS (1.9 ± 1.4° for PTAA_10_, n.s.) but showed a very strong positive correlation with the actual PTS (r at least 0.90 for the medial tibial plateau). These differences may be due to the normalization of different reference axes. In the present study, the actual PTS referring to the ATA serves as the reference for determining the deviation between the different mid‐diaphyseal axes, whereas Faschingbauer et al. [13] normalized their deviation values to the mechanical tibial axis. In lateral radiographs, the mechanical axis is posterior to the anatomical axis, so the mechanical axis per se would result in smaller slope values and, thus, greater deviation from the mid‐diaphyseal axes. Conversely, Ni et al. [33] have shown that slope measurements based on a long anatomical axis resulted in a 1.8° increase compared to those based on a short PTAA of 10 cm. These conflicting results may be explained by anterior tibial bowing, which significantly affects slope measurements with a shorter tibial anatomical axis. Recently, Hess et al. [20] have shown that with increasing tibial bowing, the PTS on short knee radiographs is underestimated compared to the PTS measured with a mechanical axis, so the PTAA method cannot be validly used in cases of excessive anterior tibial bowing.

In addition, a recent biomechanical study by Bixby et al. [6] evaluated the effect of tibial malpositioning on the radiographically measured PTS in five synthetic knee models using the measuring technique by Dejour and Bonin [11]. Malrotation of up to 15° from neutral resulted in a maximum deviation in measured PTS of 0.4°, whereas adduction and abduction of 10° or more resulted in a significant change in PTS of at least 3.4° compared to the values in the neutral position. In contrast, the present study showed that the Dejour and Bonin technique [11] was much more sensitive and reciprocal to tibial malrotation. Using the PTAA_15_ method, IE decreased the medial and lateral PTS to decrease, whereas external rotation increased the medial and lateral PTS, resulting in a maximum underestimation of 1.7° for 30° IE (*p* < .05) and a maximum overestimation of 1.8° for 30° external rotation (*p* < .01). These differences may be due to the different specimens (synthetic knee model vs. human cadaveric tibia in the present study) and test setups (free hand rotation controlled by a goniometer vs. rotary/tilting table in the present study) used in the aforementioned study, which may lead to inaccuracies in rotation control during radiographic acquisition. In addition, the present study investigated the effect of tibial malrotation on further measuring methods, such as the ATA, PTAA_10_, ATC and PTC techniques. For internal and external malrotation, a large scatter with mean deviations up to 6.1° and SDs up to 1.9° was observed between the actual PTS and these measurement methods, which was even more pronounced for varus and valgus malrotation (mean deviations up to 7.1° and SDs up to 3.3°). These findings contribute to the ongoing debate regarding the validity of slope measurements and the lack of a standardized measuring method, resulting in many clinical studies in the current literature that do not measure uniformly and are, therefore, not comparable.

However, the key concern is not the high variability in slope measurements itself, but rather that clinical decision‐making is based on the measured slope values. Particularly in the setting of multiple ACL failures, a PTS > 12° has been consistently identified as a strong predictor of recurrent instability [16, 27, 47], which is mainly attributed to excessive stress on the ACL graft [5] and graft roof impingement after ACLR [41]. Accordingly, the current literature suggests that treatment of a PTS > 12° may improve revision ACLR outcomes [7, 19, 29, 32]. However, as the indication is based on a slope value >12°, clinicians should be aware that the measuring method can significantly influence clinical decision‐making—especially if the measurement is biased by confounders. For example, in a horror scenario with an ATC‐based measurement in IR (massive overestimation) compared to a PTC‐based measurement in ER (massive underestimation), there may be an absolute difference between the PTS values of up to 10° making the decision for a slope‐reducing osteotomy too easy.

First, the actual tibial slope was assessed at the cartilaginous articular surface, whereas the radiographic tibial slope was referenced to the bony tibial plateau, so irregular cartilage thickness may have caused differences between actual and radiographically based PTS values. Nevertheless, a large number of studies have used magnet resonance [22, 25, 28] and radiographic imaging [11, 13, 26, 43, 46] to measure the tibial slope and have found no significant difference between the slope values. Second, an intramedullary steel rod served as the ATA and, thus, the reference for measuring the actual PTS, which does not account for tibial bowing. Third, the neutral position of the tibia was referenced by a perfect superimposition of the medial and lateral posterior tibial condyles, whereas the medial and lateral posterior femoral condyles are considered the main criterion of a true lateral radiograph [12, 36, 40]. Finally, inter‐ and intraobserver reliabilities were not assessed. However, several studies have reported good reliability for different radiographic‐based slope measurement techniques with intraclass correlation coefficients >0.8 [31].

## CONCLUSION

Tibial slope measurements have a high degree of variability between different measurement methods, while the ATC and PTAA methods showed the least deviation from the actual PTS measured in this in‐vitro model. Malrotation resulted in a severe distortion of the PTS values, which may alter preoperative planning and intraoperative results. Therefore, radiographic PTS measurements may be contrasted with more objective, reproducible and reliable measuring methods.

## AUTHOR CONTRIBUTIONS

**Christian Peez**: Conception and design; testing and data acquisition; statistical analysis; writing. **Caroline Waider**: Testing and data acquisition. **Adrian Deichsel**: Internal review. **Thorben Briese**: Internal review. **Lucas K. Palma Kries**: Internal review. **Elmar Herbst**: Internal review. **Michael J. Raschke**: Internal review. **Christoph Kittl**: Conception and design; testing and data acquisition; statistical analysis; writing; internal review.

## CONFLICT OF INTEREST STATEMENT

Elmar Herbst is Deputy Editor‐in‐Chief for the Knee Surgery, Sports Traumatology and Arthroscopy (KSSTA). The remaining authors declare no conflict of interest.

## SOCIAL MEDIA SUMMARY

Proximal tibial anatomical axis‐ and anterior tibial cortex‐based measurements of posterior tibial slope on lateral radiographs were found to differ least from actual posterior tibial slope, in this biomechanical investigation performed at @UK Muenster.

## ETHICS STATEMENT

The knee specimens were dissected and biomechanically tested with the permission of the Institutional Ethics Committee (File number 2022‐198‐f‐S).

## Data Availability

Data are available from the corresponding author upon reasonable request.
